# High C-Factor Cavities: How Do “Snowplow Technique”, Adhesive Application Mode and Aging Influence the Microtensile Bond Strength to Dentin?

**DOI:** 10.3290/j.jad.b4835909

**Published:** 2024-01-15

**Authors:** Robert Tee, Kirstin Vach, Nadine Schlueter, Silke Jacker-Guhr, Anne-Katrin Luehrs

**Affiliations:** a Doctoral Student, Department of Conservative Dentistry, Periodontology and Preventive Dentistry, Hannover Medical School, Hannover, Germany. Performed all experiments, wrote the manuscript, created the tables.; b Statistician and Research Associate, Department of Conservative Dentistry, Periodontology and Preventive Dentistry, Hannover Medical School, Hannover, Germany. Performed the statistical analysis, created the figures, co-wrote the results section, proofread the manuscript.; c Professor and Department Head, Department of Conservative Dentistry, Periodontology and Preventive Dentistry, Hannover Medical School, Hannover, Germany. Proofread the manuscript, contributed substantially to discussion.; d Research Associate, Department of Conservative Dentistry, Periodontology and Preventive Dentistry, Hannover Medical School, Hannover, Germany. Supervised the experiments in part, proofread the manuscript.; e Professor, Department of Conservative Dentistry, Periodontology and Preventive Dentistry, Hannover Medical School, Hannover, Germany. Research idea and experimental design, supervised the experiments in part, co-wrote and proofread the manuscript, created the figures.

**Keywords:** class-I cavity, snowplow technique, bulk filling, µTBS, configuration factor

## Abstract

**Purpose:**

To investigate the microtensile bond strength (µTBS) to dentin in class-I cavities using different layering techniques, adhesive application modes, and aging.

**Materials and Methods:**

150 caries-free human molars were randomly assigned to 8 experimental and 2 control groups (n=15 teeth/ group). For each tooth, a standardized class-I cavity was prepared (4x4x4 mm) and pretreated with a universal adhesive (self-etch or etch-and-rinse mode). Incrementally layered restorations served as the control. In the experimental groups, either lining with bulk-fill flowable composite and a layering technique, bulk filling, or the snowplow technique with one or two layers of viscous composite were applied. Four microsticks were obtained from each cavity. Half were tested initially and the other half after aging (thermocycling, 15,000 cycles, 5-55°C, n=30 sticks/group). Tobit regression was used for analyzing group differences, including analysis of interactions, Pearson’s chi-squared test or Fishers’s exact test for fracture analyses (significance level 0.05).

**Results:**

Regression analysis showed significant differences in µTBS between groups initially and after aging. In both etching modes, lining with a bulk-fill flowable composite and layering technique achieved the highest µTBS both initially and after aging. In contrast to the etching mode (self-etch < etch-and-rinse), aging did not influence µTBS significantly. The predominant failure types were adhesive and mixed, with a significantly lower number of pre-test failures in the etch-and-rinse groups.

**Conclusion:**

The etch-and-rinse mode achieves higher µTBS in class-I cavities compared to the self-etch mode. The lining technique with bulk-fill flowable composite as well as the snowplow technique yielded the highest µTBS after aging, whereas bulk filling and its combination with the snowplow technique resulted in lower µTBS.

When restoring a class-I cavity, the application technique of the restorative material is a significant treatment step. Therefore, it is crucial for the practitioner to know which resin composite and which application technique should be used in order to achieve stable long-term results. After the development of the enamel etching technique,^13^ adhesive bonding to dentin still represents one of the main challenges to a lasting bond between the tooth and composite resin materials.^2,11,17,53^ In the clinical situation, impaired adhesion is a factor in the development of recurrent caries, and thus may lead to the failure of the restoration.^36,41,52^ The bond to enamel and dentin has to withstand the polymerization stress that builds up during the polymerization of the composite due to the crosslinking of the matrix monomers into polymer chains, which is associated with volume shrinkage.^9,16^ This stress can lead not only to debonding^22,58^ but also to deflection of the tooth cusps and is associated with fractures.^34^ During polymerization, the composite consistency changes from a viscous-plastic to a rigid-elastic (gel) phase, in which no more material can flow from the free surface. The ongoing polymerization reaction causes the material to shrink and leads to stress at the interfaces.^16^

Newly developed bulk-fill resin composites are characterized by reduced shrinkage stress, which can be reached by delaying the gel point.^31^ The material changes are also achieved by additional components, such as new photoinitiators,^27^ higher translucency^12,66^ and special matrix monomers.^31^ In addition, bulk-fill composites seem to be less technique sensitive to work with.^32^ Therefore, significantly thicker layers can be placed into the cavity. This has been confirmed in many in-vitro and clinical long-term studies for a layer thickness of 4 mm.^24,29,49,64^ Polymerization should be performed carefully, as a longer curing time has a positive effect on the polymerization properties, such as degree of conversion, Vickers hardness, and polymerization shrinkage stress.^69^ In addition, the cavity design has a significant influence on the polymerization stress, which is described by the C-factor (configuration factor).^18,43^ The C-factor is the ratio of the bonded to the free surface of the restorative material.^21^ A high C-factor in a class-I cavity leads to a severe decrease in microtensile bond strength of composite compared to bonding to a flat surface.^54,67^ To counteract this effect, the incremental layering technique, in which each increment has a larger free surface area for stress relief compared to bulk filling, has been developed.^46^ Another approach to improve the integrity of the filling is to add a small layer of flowable composite as a liner. However, no clear recommendation for or against this procedure can be found in the literature.^1,8,10,14,37,62^ A third option, which attempts to compensate for the negative effects of polymerization stress through the application technique and therefore to achieve a high bond strength at the interfaces, involves the “snowplow technique.” In this technique, a flowable composite is placed on the bottom of the cavity and an additional viscous composite is placed in the cavity without prior curing, adapted, and then cured together with the flowable material.^45^ Studies have shown that this technique leaves fewer voids and fewer gaps in the marginal area.^44,49^ In contrast, there are also studies showing no additional benefit in terms of microtensile bond strength and clinical success rates of this technique.^7,42,43^ Apart from the application technique, the etching mode of the applied universal adhesive (etch-and-rinse or self-etch) can lead to different bond strengths when using the same composites.^40^ The aging of composite and the hybrid layer by methods such as thermocycling or long-term water storage is also a factor which influences the adhesive properties of the material.^59,60^

In previous studies, the snowplow technique was investigated with conventional flowable and viscous composites for gap formation and voids.^7,42,45,51^ However, the bond strength of the snowplow technique in combination with bulk-fill composites has to date not been investigated. Therefore, the aim of this study was to compare the dentin bond strength achieved by the snowplow technique using a bulk-fill flowable composite in combination with bulk-filling and conventional layering technique in standardized class-I cavities vs other conventional filling methods (layered, bulk, lining).

The null hypotheses stated that the bond strengths would not differ between (1) the investigated procedures, (2) the self-etch and the etch-and-rinse approach, and (3) the values measured initially and after thermocycling.

## Materials and Methods

### Specimen Preparation

**Fig 1:**
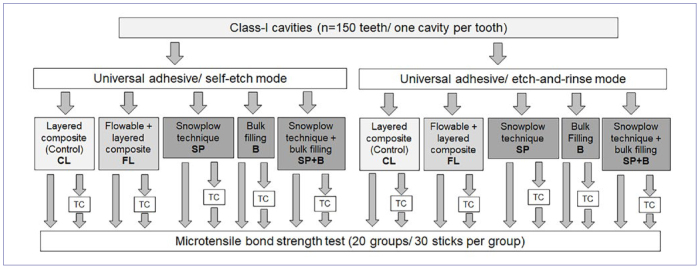
Graphic illustration of the experimental design with control and test groups as well as their respective group code. CL: control; FL: flowable + layered composite; SP: snowplow technique; B: bulk filling; SP + B: snowplow technique + bulk filling; TC = thermocycling.

For this in-vitro study, 150 permanent caries- and restoration-free human molars were collected and cleaned of debris. The teeth were stored in chloramine-T solution (0.5%) at 4°C until preparation, for no longer than 3 months. All procedures performed in this study were in accordance with the ethical standards of the institutional research committee and the 1964 Helsinki declaration and its later amendments. The use of extracted human teeth for bond strength testing was approved by the responsible ethics committee of the Hannover Medical School (no. 2092-2013). All donors gave verbal consent for anonymous use of their teeth for laboratory studies. The teeth were randomly assigned to the respective experimental and control groups (Fig 1 and Table 1).

**Table 1:** Group codes and treatment procedures for the control and test groups

Group codes	Treatment procedure
CL (control)	Packable composite layered in 2 mm horizontal increments, separate light curing of each increment
FL (flowable + layered composite)	1 mm lining with bulk-fill flowable composite, light curing, packable composite layered in 1.5 mm horizontal increments, separate light-curing of each increment
SP (snowplow technique)	First layer: 1 mm lining with bulk-fill flowable composite and a 1.5 mm horizontal increment of packable composite, simultaneous light-curing of both materials Second layer: packable composite in a 1.5 mm horizontal increment, light curing
B (bulk filling)	Bulk filling of the whole cavity with packable bulk-fill composite and light curing
SP+B (snowplow technique + bulk filling)	1 mm lining with bulk-fill flowable and bulk-filling of the cavity with packable bulk-fill composite, simultaneous light-curing of both materials

The materials and their application according to manufacturer’s instructions are shown in Table 2.

**Table 2:** Materials used in this study, their compositions and instructions for use (IFU)

Material	Manufacturer	Composition	Batch No.	IFU
Scotchbond Universal Etchant	3M Oral Care	Water, phosphoric acid, amorphous silica, polyethylene glycol, aluminium oxide	Lot 7583983 REF 41294	Etch the cavity for 15 s, then rinse thoroughly
Scotchbond Universal Plus		2-propenoic acid, 2-methyl-, diesters with 4,6-dibromo- 1,3-benzenediol 2-(2-hydroxyethoxy)ethyl 3- hydroxypropyl diethers, 2-hydroxyethyl methacrylate, 2-propenoic acid, 2-methyl-, reaction products with 1,10-decanediol and phosphorus oxide (P_2_O_5_), 2-propenoic acid, 2-methyl-, 3-(triethoxysilyl)propyl ester, reaction products with silica and 3-(triethoxysilyl)- 1-propanamine, ethanol, water, synthetic amorphous silica, fumed, crystalline-free, methacrylic acid, 3-(triethoxysilyl)propyl ester, camphorquinone, copolymer of acrylic and itaconic acid, n,n-dimethylbenzocaine, (3-aminopropyl)triethoxysilane, diethylene glycol dimethacrylate, acetic acid, copper(2+) salt, monohydrate	Lot 7675730	Self-etch: apply adhesive, rub for 20 s, air thin for 5 s, light cure for 10 s. Etch and-rinse: etch dentin for 15 s, rinsei with water, dry moderately by air blowing. For application and curing, see self-etch approach
Filtek One Bulk Fill (A1)		Silane-treated ceramic, aromatic urethane dimethacrylate, UDMA, ytterbium fluoride, silane-treated silica, 1,12-dodecane dimethycrylate, silane-treated zirconia, water Filler content (in % by volume): 58.5 Color: A1	Lot NC92613 REF 4867A1	Bulk-fill the cavity directly from the capsule and light cure for 40 s from the top surface
Z100 MP Restorative (A1)		Ceramic material, hydrolysis product with 3- (trimethoxysilyl)propyl methacrylate, 2,2’-ethylenedioxy diethyl dimethacrylate, bisphenol-A-diglycidyl methacrylate, 2-benzotriazolyl-4-methylphenol filler content (in % by volume): 66 Color: A1	Lot NE09304 REF 3022A1	Apply composite in max. 2-mm increments, light cure for 40 s after each increment
SDR flow +	Dentsply Sirona; Bensheim, Germany	Urethane dimethacrylate resin, ytterbium trifluoride, ethoxylated bisphenol-A dimethacrylate, 2,2’-ethylenedioxydiethyl dimetharcylatel, propylidynetrimethyl trimethacrylate Filler content (in % by volume): 47.4 Color: Universal	Lot 00062439 REF 60603040	Lining: Apply a 1-mm-thick layer of flowable and light cure for 20 s Snowplow: Apply a 1-mm-thick layer of flowable, apply the viscous composite, simultaneously light cure for 40 s

Each material was tested in self-etch and etch-and-rinse mode. For specimen preparation, the teeth were embedded in gypsum parallel to the tooth axis 1 mm below the cementoenamel junction. Before further preparation, a universal adhesive was applied in self-etch mode (Scotchbond Universal Plus, 3M Oral Care; St Paul, MN, USA) to the occlusal surface of the teeth, followed by placement of a flowable composite (Estelite Universal flow, Tokuyama, Tokyo, Japan) between the cusps in order to create a flat surface according to van Ende et al.^66^ Afterwards, standardized class-I cavities (4x4x4 mm) were prepared under constant water cooling with a copy-milling machine developed by the research and development laboratory of the Hannover Medical School.

A diamond bur (FG 837 314 014, Komet; Lemgo, Germany) was used for cavity preparation and was replaced after preparation of 5 cavities. Immediately after preparation, the adhesive pretreatment of the cavities was performed according to the group assignment described in Fig 1 and Table 1. The universal adhesive was applied according to the manufacturer’s instructions. In the self-etch group, the adhesive (Scotchbond Universal Plus, 3M Oral Care) was applied immediately after preparation and drying of the cavity, and then polymerized for 10 s. In the etch-and-rinse group, the whole cavity was etched with a phosphoric acid (Scotchbond Universal Etchant, 3M Oral Care) for 15 s and rinsed with a water spray for 10 s. The adhesive was then applied, dried, and polymerized for 10 s (see Table 2). In the control groups, the restoration of the cavity was performed using the incremental layering technique with 2-mm-thick composite increments (Z100 MP Restorative, shade A1, 3M Oral Care), which were each polymerized for 40 s with an LED polymerization unit (Bluephase G2, Ivoclar Vivadent; Schaan, Liechtenstein, light intensity > 1000 mW/cm^2^). The light output of the lamp was checked with a measuring device (Bluephase Meter, Ivoclar Vivadent) before placement of each new restoration.

The described polymerization protocol applies for all groups independent of the layering technique, except for the lining groups, in which the flowable composite was used as a separate liner (FL, here: polymerization for 20 s only). In the bulk-filling group, restoration was performed with a single 4-mm-thick increment with a packable bulk-fill composite (Filtek One Bulk Fill, shade A1, 3M Oral Care). In the lining groups, the cavity lining (1 mm thick) was performed using a bulk-fill flowable composite (SDR flow, Dentsply-Sirona; Bensheim, Germany). In the snowplow-technique groups, the bulk-fill flowable composite was applied in a 1-mm-thick layer, which was initially not polymerized. A 1.5-mm-thick increment of the more viscous composite material was placed on the unpolymerized flowable, and both layers were polymerized simultaneously. The cavity was then completely filled with another 1.5-mm-thick increment and polymerized. For the snowplow/bulk-filling group, the procedure was similar, but the bulk material was placed in a 4-mm-thick increment. In all experimental groups, the packable material was injected from the compule and applied in horizontal increments.

### Microtensile Bond Strength Measurement and Fracture Mode Analysis

After specimen preparation and filling procedures, teeth designated for the microtensile bond strength (µTBS) test were cut with a low-speed saw (Isomet Low Speed Cutter, Buehler; Lake Bluff, IL, USA). Each tooth was sectioned with 3 cuts in the x- and y-directions to obtain 4 microsticks, resulting in 60 sticks/treatment procedure. Half of the sticks (n = 30 per group) were tested after 24 h storage in distilled water at 37°C by μTBS, and the other half of the sticks (n = 30 per group) were aged by thermocycling (15,000 cycles, dwell time 30 s, transfer time 10 s, 5°C/55°C), and analyzed after aging. The bonded area (mm^2^) of all sticks was measured with a digital gauge before testing. During testing, the sticks were loaded (crosshead speed 0.5 mm/min) in a universal testing machine (MTD-500 plus, SD Mechatronik; Feldkirchen-Westerham, Germany) until failure to determine the μTBS. The maximum measured force (N) per stick was documented and divided by the bonded area (mm^2^) to calculate the bond strength in MPa.

After microtensile testing, all specimens were examined by light microscopy at 40X magnification (Stemi SV 6, Zeiss; Jena, Germany) to determine their fracture patterns (adhesive at the interface, cohesive in dentin or composite, mixed).

### Statistical Analysis

The sample size calculation indicated that with 30 samples per group, a group difference of 5 MPa could be detected with a power of 80% at a significance level of 5%. Sticks that fractured during sectioning or thermocycling (“zero bonds”) were included in the statistics. For this purpose, the lowest value within a group was divided by 2 and assigned to the corresponding specimens.^4^ To account for this particular distribution, which often leads to large standard deviations, Tobit regression models were used with the zero bond values as lower bounds. Fractures that occurred due to manipulation errors or more than 2 mm away from the interface in the dentin or composite were excluded from the statistical analyses.

Data were statistically analyzed with the statistical software STATA (Version 17.0; College Station, TX, USA), and the significance level was set to 0.05. For descriptive analysis, means and standard deviations were computed. For comparison of µTBSs regarding different etching mode, restoration techniques, and aging conditions, Tobit regression models with the zero-bond values as lower bound were used. In subsequent pairwise comparisons, a correction was made for multiple testing according to Scheffé. The results of the fracture analyses were analyzed with the Pearson’s chi-squared test or Fishers’s exact test, in the case of subgroup analyses.

## Results

The Tobit regression showed significant differences between the groups, both initially and after aging (p < 0.0001). In particular, significant differences (p < 0.0001) were found between self-etch and etch-and-rinse mode for all groups. Thermocycling resulted in a significantly lower bond strength only in the SP+B group in SE mode (p = 0.009).

### Self-etch Mode

**Fig 2:**
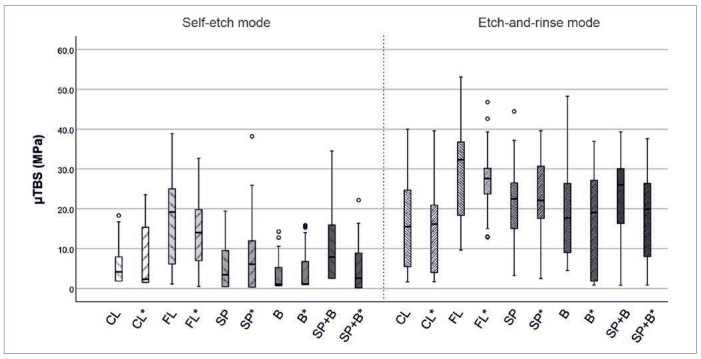
Boxplot diagram of the control and experimental groups in self-etch and etch-and-rinse mode before and after aging, with median, the 2nd and 3rd quartile as well as minimum/maximum values, the small circles display outliers. CL: control; FL: flowable + layered composite; SP: snowplow technique; B: bulk filling; SP+B: snowplow technique + bulk filling. * aged by thermocycling.

The results of the µTBS test are shown in Table 3 and Fig 2. Within the self-etch groups, the highest initial µTBS was achieved by the lining technique (FL: 16.8 ± 11.1 MPa), which was significantly higher compared to the layered control (CL: 6.1 ± 5.4, p < 0.001), the bulk-fill (B: 3.2 ± 3.8 MPa, p < 0.001), and the snowplow group (SP: 5.6 ± 6.1 MPa, p = 0.001). Also, the use of the snowplow technique in combination with bulk filling elevated the µTBS significantly when compared to bulk-fill only (SP+B vs B: 9.9 ± 8.7 MPa vs 3.2 ± 3.8 MPa, p = 0.005). After thermocycling, the lining group (FL: 14.7 ± 9.4 MPa) still exhibited the highest µTBS, which was not statistically significantly different from the snowplow group (SP: 8.1 ± 9.3 MPa, p = 0.117), but it was compared to the layered control (CL: 7.7 ± 7.7 MPa, p = 0.045), the bulk group (B: 4.7 ± 5.3 MPa, p < 0.001), and the snowplow/bulk-fill group (SP+B: 5.4 ± 6.4 MPa, p < 0.001).

**Table 3:** Results of the µTBS test and the fracture analyses before and after aging with means and standard deviations (in MPa), total number of samples, zero bonds, samples excluded from statistics. Groups with the same letters are not statistically significantly different regarding comparisons within the same aging and etch mode.

	Self-etch					Etch-and-rinse				
	CL	FL	SP	B	SP+B	CL	FL	SP	B	SP+B
24-h water storage	6.1 (± 5.4)^ab^	16.8 (± 11.1)^c^	5.6 (± 6.1)^ab^	3.2 (± 3.8)^a^	9.9 (± 8.7)^bc^	16.1 (± 11.1)^a^	29.6 (± 11.7)^b^	20.8 (± 10.0)^a^	18.3 (± 11.7)^a^	22.4 (± 11.0)^ab^
n/zero bonds/samples excluded from statistics	30/13/2	30/3/1	30/12/1	30/15/0	30/12/0	30/6/2	30/0/1	30/2/1	30/7/0	30/2/0
Aging by thermocycling for 15,000 cycles	7.7 (± 7.7)^a^	14.7 (± 9.4)^b^	8.1 (± 9.3)^ab^	4.7 (± 5.3)^a^	5.4 (± 6.4)^a^	15.1 (± 11.3)^a^	27.5 (± 7.7)^b^	22.7 (± 11.5)^ab^	16.5 (± 11.9)^a^	18.2 (± 11.4)^a^
n/zero bonds/samples excluded from statistics	30/15/0	30/2/1	30/12/0	30/16/2	30/11/0	30/6/0	30/0/0	30/3/1	30/6/0	30/2/0

Group codes: CL: control; FL: flowable + layered composite; SP: snowplow technique; B: bulk filling; SP+B: snowplow technique + bulk filling.

### Etch-and-rinse Mode

Within the etch-and-rinse groups, the lining group (FL) again showed the highest µTBS initially (29.6 ± 11.7 MPa) and after aging (27.5 ± 7.7 MPa). Before aging, the bond strength of this group was significantly higher than the layered control (CL: 16.1 ± 11.1 MPa, p = 0.001) and the bulk-fill group (B: 18.3 ± 11.7 MPa, p = 0.008). After thermocycling, the µTBS of the lining group was comparable to the snowplow group (SP: 22.7 ± 11.5 MPa), but was significantly higher compared to the snowplow/bulk-fill group (FL vs SP+B: 18.2 ± 11.4 MPa, p = 0.013; FL vs CL: 15.1 ± 11.3 MPa, p < 0.001, FL vs B: 16.5 ± 11.9 MPa, p = 0.003). There were no statistically significant differences between the other groups.

### Overall Analyses

In addition, an interaction analysis was carried out to more precisely quantify the individual effects of etching mode, restoration technique, and aging.

Overall, the statistical analyses showed significantly lower µTBS for the self-etch application compared to the etch-and-rinse mode (Δ12.9 MPa, p < 0.001). Aging did not affect the bond strength (Δ0.6 MPa, p = 0.446). Regarding the type of restoration, the lining technique (FL) exhibited significantly higher bond strengths than did all other techniques (CL, SP, B and SP+B, all p < 0.001). The largest observed difference was 11.6 MPa, between the lining technique and the control, which was smaller than the difference between the etching modes. The results of the snowplow technique were comparable to all application methods apart from the above-mentioned lining group (FL).

### Fracture Analyses

**Fig 3:**
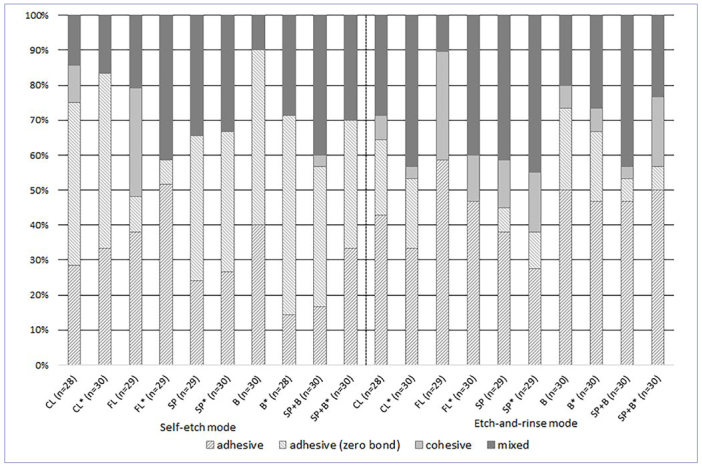
Graphic illustration of the fracture modes for the control and test groups, differentiated by failure mode. The total number of adhesive failures are the result of the modes “adhesive” + “adhesive (zero bond)”; both are displayed as hatched columns. CL: control; FL: flowable + layered composite; SP: snowplow technique; B: bulk filling; SP+B: snowplow technique + bulk filling.

Overall, the predominant failure types initially and after thermocycling were adhesive (63.0 vs 61.1 %) and mixed fractures (26.4 vs 32.8 %). When comparing the initial vs the aged groups in terms of their fracture patterns, no significant differences were found (p = 0.057). When comparing the fracture modes according to the etching modes (self-etch vs etch-and-rinse, p < 0.001), a larger amount of cohesive fractures occurred in the etch-and-rinse groups. When all groups were analyzed separately regarding the etching modes and aging procedures, the fracture types initially differed significantly in self-etch mode (p < 0.001) and etch-and-rinse mode (p = 0.005), but not after thermocycling (p = 0.340 vs p = 0.214). The fracture modes per groups are displayed in Fig 3. In total, fewer zero bonds occurred in the etch-and-rinse groups than in the self-etch groups (p < 0.001, see Table 3 and Fig 3).

## Discussion

In clinical practice, class-I cavities represent a major challenge to the restorative material due to their high C-factor, which is associated with increased polymerization stress. In this study, the microtensile bond strength (µTBS) test was used to investigate whether the application of composites using the snowplow technique offers advantages in terms of adhesion to dentin in class-I cavities compared with the incremental layering, bulk-filling or lining technique, and if the application mode of the universal adhesive influences the adhesion values.

The first null hypothesis has to be rejected, as the bond strengths of the investigated procedures differed significantly from each other. The lining technique showed the highest bond strengths to dentin in both the self-etch and etch-and-rinse groups, with significant differences depending on the etching mode and aging by thermocycling. Previous studies have shown that the use of a flowable composite as a liner results in less microleakage and fewer voids at the interface compared to the incremental layering technique.^1,5,6,14,39,62^ In contrast, another study demonstrated that a flowable composite as a liner does not offer any advantage in stress reduction during polymerization.^10^ In addition, a systematic review reported that lining does not reduce microleakage and therefore does not improve clinical performance.^8^ The bond strength at the composite-dentin interface is significantly influenced by shrinkage stress. The shrinkage stress is the product of the volumetric shrinkage and the modulus of elasticity of the respective composite.^35^ The shrinkage is directly dependent on the filler content of the composite. A high filler content lowers the polymerization shrinkage of the composite.^65^ Thus, it can be assumed that the bulk-fill composite used in this study with a filler content of 58.5% by volume has a lower volumetric shrinkage than the flowable bulk-fill composite, which has a filler content of 47.4% by volume.^38^ On the other hand, the flowable bulk-fill composite has a significantly lower e-modulus than other composites and therefore exhibits lower shrinkage stress than viscous bulk-fill composites.^19,30,31,33^ This observation could explain the results of this study, as the bulk fill group (B) and the snowplow bulk group (SP+B) achieved significantly lower bond strengths compared to the lining group (FL). Due to the low elastic modulus of the flowable bulk-fill composite and the associated low polymerization stress, the flowable bulk-fill composite can serve as a “stress breaker” between the viscous composite and the dentin. Without a separately polymerized layer of flowable composite, the higher shrinkage stress of the large increment of viscous bulk-fill composite in the B and SP+B group seems to have a detrimental effect on the adhesive bond. Studies have shown no adverse effect of bulk-fill flowable composites even in increments up to 4 mm, compared to conventional composites layered in increments.^24,29,49,64^ The bulk-fill flowable composite thus offers the advantage of good dentin wettability^14^ and at the same time compensates the disadvantages of a conventional flowable composite – such as high polymerization shrinkage – by special material additives and increased translucency.^12^ The present study has shown that the highest bond strengths to dentin in class-I cavities can be achieved with the lining technique using a bulk-fill flowable composite (group FL), and, after aging, also with the snowplow technique (SP). This can be explained by the fact that when using the lining technique, the 4-mm-deep cavity was filled with 3 thin, separately polymerized layers, which was done in both FL and SP groups. Although the bond strength data of the above-mentioned groups showed comparable results (FL vs SP), a higher number of zero bonds were found in the snowplow groups with both etching modes. The high C-factor imposed by the cavity design, which represents a worst-case scenario in this study, and the associated polymerization shrinkage could thus be significantly reduced by placing individual layers compared to the other groups.^67^

Another explanation can be the low layer thickness of 1 mm and the high translucency of the bulk-fill flowable composite. The light generated by the curing light loses intensity due to the filler particles of the composite and the associated light scattering and absorption.^23,48^ The penetration depth for curing (DOC) the composite is therefore limited and, particularly in the area where the interface with the dentin was created, the energy for sufficient curing is lacking if the layer thickness of the composite is too high.^44^ Apart from the above-mentioned increased translucency due to a low filler content, bulk-fill composites exhibit modifications of the fillers in shape, size, and coating.^3,12^ Since translucency is directly related to DOC as discussed above, bulk-fill composites can achieve higher DOC than conventional composites.^65^ It is interesting to note that flowable bulk-fill composites achieve a higher DOC than viscous bulk-fill composites.^28,65^ This is an advantage when using a bulk-fill flowable composite for the lining technique, a procedure which achieved significantly higher adhesion values compared to the bulk or snowplow bulk-fill group in our study. The viscous bulk-fill composite was applied in a 4-mm-thick layer in these groups, which could be detrimental to the DOC at the interface with dentin,^55^ especially compared to the lining group. In the lining and snowplow groups, an additional incremental layering technique was used, which was originally invented for conventional composites in order to ensure the penetration of light and reduce the C-factor.^18,67^

As the conventional lining technique is time consuming, alternative methods for restoring a cavity were developed. In this study, the snowplow technique, in which a viscous composite is placed on a low-viscosity (flowable) composite without separate light curing and the two are cured together, was one of the methods investigated and compared with other filling methods. This filling technique is intended to combine the good wettability and homogeneous marginal adaptation of a flowable composite with the good mechanical properties of a viscous composite.^26,45^ It is worth mentioning that the bulk snowplow technique has yielded slightly better adhesion values before aging, which may be due to the lower technique sensitivity and the lower shrinkage stress. Most of the studies that have been conducted on the snowplow technique have examined microleakage and marginal integrity. However, a previous study also showed that the snowplow technique can achieve a microtensile bond strength equivalent to that of the incremental layer technique.^43^ This is consistent with the results of this study (control vs SP), where both application methods were not significantly different from each other, independent of aging or application mode.

Also, the present study showed significantly higher bond strengths for the etch-and-rinse procedure when compared to the self-etch approach. Therefore, it can be concluded that in class-I cavities, the use of the etch-and-rinse technique in conjunction with a universal adhesive results in a significant increase in bond strength to dentin and fewer pre-test failures compared to the self-etch technique, regardless of the method used. As a consequence, the second null hypothesis, that the bond strengths between the self-etch and the etch-and-rinse approach would not differ, has to be rejected. In this study, it has been demonstrated that when the bond to dentin is established using the self-etch technique, the polymerization stress is sufficiently counteracted by the lining technique. Clinically, the advantage of a universal adhesive is that it can be applied both in etch-and-rinse and self-etch mode, depending on the intraoral situation.^15^ The etch-and-rinse mode is based on the micromechanical interlocking of the adhesive in the areas where the dentin is demineralized by etching with phosphoric acid. When the universal adhesive is applied in self-etch mode, a 1-µm-deep demineralized zone is created by acidic functional monomers. In addition, a chemical bond to the calcium in the hydroxyapatite can be established by the functional monomer 10-MDP.^68^ However, this chemical bond can no longer occur after phosphoric acid etching of the dentin, since the hydroxyapatite is missing as a bonding partner, which is why selective enamel etching in conjunction with a universal adhesive is recommended.^15,50^ In the present study, higher bond strengths were nevertheless achieved by using the etch-and-rinse technique compared to the self-etch technique, and therefore, micromechanical interaction might be a more relevant factor influencing the bond strength in high C-factor cavities compared to chemical interaction. The adhesive and its application method are one of the most important factors in achieving the bond strength of the composite used, which is also one result of our study.^32^

Furthermore, the resulting hybrid layer thickness is significantly higher after acid etching compared to the self-etch application.^57^ As the hybrid layer acts as a stress absorbing zone during the bonding procedure, thicker hybrid layers, which occur during the etch-and-rinse approach, might somewhat compensate the polymerization stress in high C-factor cavities.^20,63^ In contrast, the additional chemical bond created via 10-MDP during self-etch procedures is not sufficient to compensate for the high forces of polymerization shrinkage which occurred in our study setup. If the universal adhesive is applied in self-etch mode, a very low thickness of the hybrid layer is produced after the adhesive has been dispersed. The oxygen present may impair the complete polymerization of this layer, and even makes it more susceptible to hydrolytic processes.^47^ This could be another explanation for the lower adhesion values in the self-etch groups in this study. One possible solution to overcome this problem is to apply an additional hydrophobic bonding layer after the use of the universal adhesive.^56^

Furthermore, the hybrid layer is subject to both chemical and mechanical degradation.^17^ To investigate the degradation process and simulate aging, all groups were subjected to thermocycling.^61^ Due to the higher demineralization depth of 3 to 6 μm^68^ achieved by phosphoric acid and possible incomplete adhesive infiltration, the hybrid layer generated during the etch-and-rinse application is therefore more susceptible to degradation processes, such as hydrolytic degradation and water resorption.^68^ HEMA, which is present in a high proportion in universal adhesives and also in the adhesive used in our study, has hydrophilic properties which promote water sorption and as a result hydrolytic degradation of the hybrid layer.^2^ Since significantly fewer collagen fibrils are exposed when the adhesive is applied in the self-etch process, it can be assumed that the hybrid layer generated during the self-etch process is less susceptible to hydrolysis.^25^ In our study, aging did not affect the bond strength in either application mode, and the values remained stable, although on a significantly higher level for the etch-and-rinse application. Therefore, chemical degradation seems to have had only a minor effect on the results presented here. As the bond strengths after thermocycling did not decrease significantly, the third null hypothesis, that the aging process would not influence the bond strength, was confirmed.

## Conclusion

In a high C-factor cavity, the etch-and-rinse technique led to a significantly higher bond strength when compared to the self-etch mode. The highest µTBS was achieved by the lining technique using a bulk-fill flowable composite. The snowplow technique in combination with the incremental layering technique resulted in bond strengths equal to the lining technique in both etching modes after aging, whereas bulk filling, even in combination with the snowplow technique, showed inferior results.

## Clinical relevance:

Composite restorations placed in high C-factor cavities, such as class-I cavities, benefit from an etch-and-rinse approach. The same is true for a separately applied layer of flowable composite.
